# Managerial competences of researchers from Nursing research groups*

**DOI:** 10.1590/1518.8345.4535.3445

**Published:** 2021-07-19

**Authors:** Greici Capellari Fabrizzio, Alacoque Lorenzini Erdmann, José Luís Guedes dos Santos, Susana Cararo Confortin, Ana Lúcia Schaefer Ferreira de Mello, Aida Maris Peres

**Affiliations:** 1 Universidade Federal de Santa Catarina, Florianópolis, SC, Brazil.; 2 Scholarship holder at the Conselho Nacional de Desenvolvimento Científico e Tecnológico (CNPq), Brazil.; 3 Universidade Federal do Maranhão, São Luís, MA, Brazil.; 4 Scholarship holder at the DECIT/Ministério da Saúde, Brazil.; 5 Universidade Federal do Paraná, Curitiba, PR, Brazil.; 6 Scholarship holder at the Universidade Federal do Paraná, Brazil.

**Keywords:** Research Personnel, Professional Competence, Research Groups, Competency-Based Education, Nursing Education, Nursing Education Research, Pesquisadores, Competência Profissional, Grupos de Pesquisa, Educação Baseada em Competências, Educação em Enfermagem, Pesquisa em Educação de Enfermagem, Investigadores, Competencia Profesional, Grupos de Investigación, Educación Basada en Competencias, Educación en Enfermería, Investigación en Educación de Enfermaría

## Abstract

**Objective:**

to analyze the managerial competences of researchers from research groups linked to a Graduate Program in Nursing.

**Method:**

a cross-sectional study with researchers from Nursing research groups, which analyzed the managerial competences by means of a Scale of Managerial Competences in Research Groups containing 50 items related to people management and research results (Factor 1) and resource provisioning and people management (Factor 2), with answers 4 and 5 considered as sufficient dexterity for each competence analyzed. For data analysis, logistic regression was used.

**Results:**

of the 219 participants evaluated, the prevalence was 48.86% of sufficient dexterity for factor 1 and 32.88% of sufficient dexterity for factor 2; with 41.21% of sufficient dexterity for Managerial Competences in research groups. A significant difference was identified in the proportions of the managerial competences for schooling, age group and performance in the group (p≤0.001). There were differences in mean age, time of experience with research and participation in the research group (p≤0.001), between the participants with sufficient and insufficient dexterity for the managerial competences.

**Conclusion:**

the results obtained in this study emphasize the potential of the research groups for the development of managerial competences of Nursing researchers, especially for people management.

## Abstract (pt)

**Objective:**

analisar as competências gerenciais de pesquisadores de grupos de pesquisa vinculados a um Programa de Pós-graduação em Enfermagem.

**Método:**

estudo transversal com pesquisadores de grupos de pesquisa em enfermagem, que analisou as competências gerenciais por meio de uma Escala de Competências Gerenciais em Grupos de Pesquisa contendo 50 itens relacionados à gestão de pessoas e de resultados de pesquisa (Fator 1) e captação de recursos e gestão de pessoas (Fator 2); as respostas 4 e 5 foram consideradas como domínio suficiente para cada competência analisada. Para análise de dados, utilizou-se regressão logística.

**Resultados:**

dos 219 participantes avaliados, a prevalência foi de 48,86% suficientes para o fator 1 e 32,88% suficientes para o fator 2; 41,21% suficientes para Competências Gerenciais em Grupos de Pesquisa. Identificou-se diferença significativa nas proporções das competências gerenciais para escolaridade, grupo etário e atuação no grupo (p ≤0,001). Houve diferença na média de idade, tempo de experiência com pesquisa e participação no grupo de pesquisa (p ≤0,001), entre os participantes suficientes e insuficientes para as competências gerenciais.

**Conclusão:**

os resultados obtidos neste estudo enfatizam o potencial dos grupos de pesquisa para o desenvolvimento de competências gerenciais dos pesquisadores em enfermagem, especialmente para gestão de pessoas.

## Abstract (es)

**Objetivo:**

analizar las competencias directivas de investigadores de grupos de investigación vinculados a un Programa de Posgrado en Enfermería.

**Método:**

estudio transversal con investigadores de grupos de investigación en enfermería, que analizó las competencias directivas a través de una Escala de Competencias Directivas en Grupos de Investigación que contiene 50 ítems relacionados con la gestión de personas y resultados de investigación (Factor 1) y captación de recursos y gestión de personas (Factor 2), las respuestas 4 y 5 se consideraron dominio suficiente para cada competencia analizada. Para el análisis de datos se utilizó regresión logística.

**Resultados:**

de los 219 participantes evaluados, la prevalencia fue 48,86% suficiente para el factor 1 y 32,88% suficiente para el factor 2; 41,21% suficiente para competencias directivas en grupos de investigación. Se identificó una diferencia significativa en las proporciones de competencias gerenciales por escolaridad, grupo de edad y desempeño en el grupo (p≤0,001). Hubo diferencia en la edad promedio, tiempo de experiencia con la investigación y participación en el grupo de investigación (p≤0,001), entre participantes suficientes e insuficientes para competencias gerenciales.

**Conclusión:**

los resultados obtenidos en este estudio enfatizan el potencial de los grupos de investigación para el desarrollo de competencias directivas de los investigadores de enfermería, especialmente para la gestión de personas.

## Introduction

Managerial competences are behaviors, observed or developed, expressed by means of knowledge, skills and individual attitudes that have an impact on the production of better organizational results. The synergy among these three elements positively influences individuals, teams and organizations in attaining their objectives^1^.

In Brazil, the work of researchers, whether they are professors, leaders and researchers in training since scientific initiation, undergraduates, MS students, specialization students, PhD students, post-doctoral students and technicians, within the scope of the research groups, is guided by the use of multiple competences, depending on the work developed. In order to carry out actions linked to the production of scientific knowledge, each researcher uses strategies considered adequate, at each stage of the investigative process^2^.

The academic activities developed by professors acting in the health area have undergone significant changes over the years due to changes in the educational system, the health system and society^3^. Competences that help in their training as high-impact, productive researchers, competent managers and academic leaders started to be demanded from higher education professors^4^.

These competences are aimed at meeting the demands inherent to the work of a researcher. Among them, the planning of the research process, from its conception to the dissemination of the results, proposal and debate of research topics with members of the group, management of human, financial and process resources; fundraising and establishment of partnerships are highlighted^1^.

A scoping review of Canadian researchers brought together competences for researchers in the health area, considering the following categories: knowledge, skills, and attitudes. In terms of knowledge, competences focused on research methods; participatory approach; evidence and results; cultural context; logistics; establishment of priorities and research aimed at the patients were highlighted. The skills include: communication; teamwork; project management and conflict management^5^.

The attitudes encompass personal characteristics of the researchers, such as: encouraging; supportive; welcoming; optimist; curious; and emotionally intelligent, as well as attitudes towards building partnerships and relationships; management sharing; respect for different perspectives; valuing mutual trust; support for the use of creative methodologies and flexibility in conducting the work; social contribution; respect for community values and patients’ experiences^5^.

In the academic context, research groups are unique spaces for the development of the aforementioned professional and personal competences. The act of researching stimulates critical reflection on the Nursing praxis in all its areas: care, management and teaching; and is directly related to problem solving, improvement of care processes and foundation of the praxis^6^.

Brazilian graduate programs are real transformation engines, in the social and economic spheres; they play an important role in the development of science, technology and innovation. Nursing research laboratories are linked to these programs and corroborate growth^7^.

Previous studies carried out with Nursing research groups addressed aspects mainly related to the profile and the functionalities, with a focus on the research line and concentration area of the laboratories^8^^-^ 11. In this sense, the study contributions focus on the identification of managerial competences of Nursing researchers and advances in the demonstration of new perspectives so that the research laboratories enhance the development of managerial competences in their members.

In view of the above, the guiding question of the study was: What are the managerial competencies of researchers inserted in research groups linked to a Graduate Program in Nursing? Based on this question, the objective was to analyze the managerial competences of researchers from research groups linked to a Graduate Program in Nursing.

## Method

A cross-sectional study, in which the study scenario was a public university in southern Brazil. The study population was composed of researchers from 14 Nursing research groups linked to a stricto sensu Graduate Program in Nursing.

The research groups are devoted to studies related to several areas of Nursing knowledge: health and nursing education, acute and chronic health situations, history of Nursing, health and nursing management, child health, woman and newborn health, health promotion, health technologies, health, nursing and rehabilitation work process, among others. The number of participants in each group varies from 15 to 71 members, with the collaboration of foreigners.

The survey was conducted from June to September 2018. The inclusion criteria were defined as follows: being an undergraduate and graduate professor or student, member and active in the research groups at the time of data collection, without time limitation. Researchers who were on leave of any nature during the data collection period were excluded.

A population sample calculation was performed with the intention of estimating the minimum number of participants in the research. The following data are considered: total population of participants in the 14 research laboratories = 498; confidence level = 95%; sampling error = 5%. Thus, a sample (n) of 217 researchers^12^ was estimated.

For the selection of the participants, non-probabilistic sampling was used, for convenience, as data collection was developed with all participants present at the research laboratory meetings. Thus, a sample of 219 members of research groups was obtained.

In the stage that preceded data collection, the instrument underwent a pilot study, for face and content validity, by two PhD students experienced in the theme in question, who were not participants in the research. This stage aimed to identify possible difficulties in completing the instrument. However, there was a consensus on the easy understanding of filling in the instrument, with no changes needed.

The data were collected using an instrument with two parts: 1) Characterization of the researchers, with questions regarding the sociodemographic profile and performance in the group; and 2) Scale of Managerial Competences in Research Groups.

The dependent variables were the 50 items of the Scale of Managerial Competences in research groups with seven categories (0 - no dexterity; 1; 2; 3; 4; 5 - complete dexterity; and NA - Not Applicable). The independent variables were the following: age group, with age being collected in a gross manner and later classified into five categories (<25; 26-35; 36-45; 46-55; >56 years old); gender (female, male); training (others, Nursing); schooling level with six categories (incomplete higher education; complete higher education; complete specialization; complete master’s degree; complete PhD; post-doctorate); type of performance in the group with six categories (leader/researcher/professor; MS/PhD; graduation; other participants; residency; post-doctorate); time of research experience in years and time of participation in the research group in years.

The Scale of Managerial Competences in Research Groups is Brazilian, it was developed and validated in the University of Brasília. It consists of 50 items and is divided into two factors, as shown in Figure 1 1.

**Figure 1:** Definition for the factors related to Managerial Competences

Factors	Items	Definition
Factor 1 - People and research results management	41	Set of managerial competences related to team management, which interact in order to achieve the results derived from the group's research activities and projects. Examples: setting deadlines, organizing meetings, encouraging communication and motivation ability
Factor 2 - Fundraising and people recruitment	09	Set of managerial competences aimed at supplying resources, attracting researchers, and collaborating with experts in the group's research activities and projects. Examples: establishment of partnerships between companies and researchers, search, and management of resources

The data were collected during the meetings of the research groups through previous contact with their leaders. The self-administered instrument was distributed by the responsible researcher and by a duly trained technical team. The mean time to fill in the instrument was 15 minutes.

The Likert-type scale has dichotomous variables referring to the items on the scale. Thus, the score is assigned according to the researcher’s degree of dexterity in relation to a given competence (0 - no dexterity; 1; 2; 3; 4; 5 - complete dexterity; and NA - Not Applicable). Scores 0, 1, 2 and 3 were considered as insufficient dexterity and scores 4 and 5 as sufficient dexterity for that managerial competence^13^.

Data analysis was performed using the IBM SPSS Statistics® 20.0 for Windows statistical program. For all the variables, descriptive analyses were performed. For the quantitative variables, the difference of the means between the managerial competencies was verified using the Student’s t-test. For the qualitative variables, the proportion was calculated, as well as the difference between the occurrence of the managerial competencies for the independent variables, using Fisher’s Exact or Chi-Square tests. Variables with a p-value ≤0.05 in the bivariate analysis were considered statistically significant. The gross analysis was performed using logistic regression and the gross *Odds Ratio* (OR) was estimated, with its respective Confidence Intervals (95% CI). The missings were readjusted, that is, the information not answered was readjusted for the number of non-respondent participants. For this reason, the total number of participants varies between the variables.

Regarding the ethical aspects, the research was carried out in accordance with Resolution No. 466/2012 of the National Health Council. The study was approved by the Committee of Ethics in Research with Human Beings, under opinion number 2,595,322. All the participants were informed about the research objectives and signed the Free and Informed Consent Form (FICF).

## Results

Of the 219 study participants, the majority were female (89.5%, n=196), aged between 26 and 35 years old (35.8%, n=78), had completed a master’s degree (25.5%, n=88), undergraduate Nursing (96.3%, n=206) and acted in the group as an MS/PhD student (40.2%, n=88). The mean time of experience in research was approximately 8 years (SD: 7.50; 0-38) and of experience in the research group, 4 years (SD: 5.72; 0-31). Table 1 characterizes the sample by means of the sociodemographic and professional profile of the study participants.

**Table 1:** Sociodemographic and professional profile of the participants in research groups. Southern Brazil, 2018

Variables	n* (%)^†^	AM^‡^SD^§^
Gender (n*=219)		
Female	196 (89.5)	
Male	23 (10.5)	
Schooling (n*=216)		
Incomplete higher education	53 (24.5)	
Complete higher education	28 (13.0)	
Complete specialization	40 (18.5)	
Complete master's degree	55 (25.5)	
Complete PhD	26 (12.0)	
Post-Doctorate	14 (6.5)	
Training (n*=214)		
Others	8 (3.7)	
Nursing	206 (96.3)	
Age group (n*=218)		
Up to 25 years old	65 (29.8)	
26 to 35 years old	78 (35.8)	
36 to 45 years old	47 (21.6)	
46 to 55 years old	18 (8.3)	
56 years old or more	10 (4.6)	
Performance in the group (n*=219)		
Leader/Researcher/Professor	37 (16.9)	
Master's degree/PhD	88 (40.2)	
Graduation	58 (26.5)	
Other participants	31 (14.2)	
Residence	3 (1.4)	
Post-Doctorate	2 (0.9)	
Age (years old)	218	33.21 10.36
Research experience (years)	211	8.06 7.50
Participation in the research group (years)	219	4.18 5.72

* n = Total number of participants;

† (%) = Percentage;

‡ AM = Arithmetic Mean;

§ SD = Standard Deviation

Table 2 shows the participants with insufficient and sufficient dexterity in relation to the factors and the managerial competences in research groups. A larger number of participants with sufficient dexterity for Factor 1 - People and research results management was identified.

**Table 2:** Participants with insufficient and sufficient dexterity for Factors 1 and 2, and for the managerial competences (n*=219). Southern Brazil, 2018

	n*	%^†^
Factor 1 - People and research results management		
Insufficient	112	51,14
Sufficient	107	48,86
Factor 2 - Fundraising and people recruitment		
Insufficient	147	67,12
Sufficient	72	32,88
Managerial competences in research groups		
Insufficient	120	54,79
Sufficient	99	41,21

* n = Total number of participants;

† (%) = Percentage

Table 3 shows the association between the sociodemographic and professional variables in view of the managerial competences in research groups. There was a statistically significant difference in the proportions of managerial competences for schooling, age group and performance in the group. In addition, there was a difference in mean age, time of experience with research, and participation in the research group between participants with sufficient and insufficient dexterity for managerial competences.

**Table 3:** Association between the sociodemographic and professional variables in view of the managerial competences of the participants in research groups. Southern Brazil, 2018

	Insufficient		Sufficient		p-valor^‡^
	n*	%^†^	n*	%^†^	
Gender					0,478
Female	109	90,83	87	87,88	
Male	11	9,17	12	12,12	
Schooling					≤0,001
Incomplete higher education	41	34,75	12	12,24	
Higher education	15	12,71	13	13,27	
Specialization	28	23,73	12	12,24	
Master's degree	30	25,42	25	25,51	
PhD	3	2,54	23	23,47	
Post-Doctorate	1	0,85	13	13,57	
Training					0,787
Others	4	3,42	4	4,12	
Nursing	113	96,58	93	95,88	
Age group					≤0,001
Up to 25 years old	46	38,66	19	19,19	
26 to 35 years old	47	39,50	31	31,31	
36 to 45 years old	22	18,49	25	25,25	
46 to 55 years old	4	3,36	14	14,14	
56 years old or more	0	0	10	10,1	
Performance in the group					≤0,001
Graduation	43	36,13	13	13,13	
MS/PhD/Res.^§^	54	45,38	40	40,40	
Prof./Leader/Post-Doc^||^	4	3,36	33	33,33	
Other participants	18	15,13	13	13,13	
Age (years old)	119	29,95 (7,45)	99	37,13 (11,92)	≤0,001
Research experience (years)	118	5,21 (4,62)	93	11,68 (8,80)	≤0,001
Participation in the group (years)	120	2,32 (2,80)	99	6,44 (7,33)	≤0,001

* n = Total number of participants;

† (%) = Percentage;

‡ p-value = Chi-Square Test;

§ MS/PhD/Residency;

|| Professors/Leaders/Post-PhDs

In the gross analysis, in relation to the managerial competences, the individuals who had completed higher education, completed master’s degree, completed PhD, and completed post-doctorate were, respectively, 2.96 (85%CI: 1.11-7.90), 2.84 (95%CI: 1.24-6.55), 26.19 (95%CI: 6.69-102.49) and 44.41 (95%CI: 5.26-374.98) times more likely to have managerial competences when compared to the participants with incomplete higher education degrees.

As for the age group, the participants aged 36 to 45 years old and 46 to 55 years old were, respectively, 2.75 (95%CI: 1.26-6.02) and 8.47 (95%CI: 2.47-2.08) times more likely to have sufficient managerial competences when compared to individuals up to 25 years old. In the performance in the research group, MS, PhD and residency students were 2.45 (95%CI: 1.65-5.15) and professors, leaders and postdoctoral students were 27.29 (95%CI: 8.14-91.42) times more likely to have sufficient managerial competences when compared with undergraduate students.

The data showed an 8% increase (OR:1.08; 95%CI: 1.04-1.11) in the chance of presenting sufficient managerial competences for each year of increase in age. Regarding the time of experience with research, the chance of having sufficient managerial competences increases 16% with each year of experience in research (OR:1.16; 95%CI: 1.10-1.23). When participating in a research group, the chance of having sufficient managerial competences increases 18% (OR:1.18; 95%CI: 1.10-1.27) per year.

**Table 4:** Gross analysis in relation to the factors associated with the managerial competences of participants in research groups. Southern Brazil, 2018

	Sufficient		
	OR*	95% CI	p-valor
Gender			0,479
Female	1		
Male	1,37	0,57-3,25	
Schooling			≤0,001
Incomplete higher education	1		
Higher education	2,96	1,11-7,90	
Specialization	1,46	0,57-3,72	
Master's degree	2,84	1,24-6,55	
PhD	26,19	6,69-102,49	
Post-Doctorate	44,41	5,26-374,98	
Training			0,787
Others	1		
Nursing	0,82	0,20-3,38	
Age group			≤0,001
Up to 25 years old	1		
26 to 35 years old	1,59	0,79-3,22	
36 to 45 years old	2,75	1,26-6,02	
46 to 55 years old	8,47	2,47-2,08	
56 years old or more	-		
Performance in the group			≤0,001
Graduation	1		
MS/PhD/Residency	2,45	1,65-5,15	
Professors/Leaders/Post-Doc	27,29	8,14-91,42	
Other participants	2,39	0,93-6,15	
Age (years old)	1,08	1,04-1,11	≤0,001
Research experience (years)	1,16	1,10-1,23	≤0,001
Participation in the research group (years)	1,18	1,10-1,27	≤0,001

* OR = Odds Ratio

## Discussion

The results obtained in this study emphasize the potential of the research groups for the development of managerial competences of Nursing researchers, especially for people management. As far as it is known, this was the first study that analyzed the managerial competences of researchers in Nursing research groups in Brazil.

The sociodemographic profile was mostly composed of females, a fact that shows a high percentage of female researchers inserted in postgraduate research groups in Nursing. Although it hinders the establishment of statistical patterns and the analysis of comparative data between genders and other variables, this data was not considered a limitation of the study, as it represents a characteristic of Nursing, in which 85.1% of the professionals are female^14^.

Among the two factors expressed in the scale, the participants demonstrated a greater degree of sufficiency for Factor 1, related to people and research results management. This result can be related to the training of nurses, which aims at developing managerial competences with a focus on leadership and people management in line with the National Curriculum Guidelines for the Undergraduate Nursing Course in Brazil^15^.

The emphasis on the development of qualitative research studies in Nursing may also have contributed to this result^16^. This type of research is based on the collection of verbal and written data, for example, through interviews and documentary analysis. Such techniques do not require the purchase and use of technological supplies and/or resources, nor the composition of large research teams.

On the other hand, although this is the factor that reached the largest number of participants with sufficient dexterity, it is important that Nursing professors improve their skills belonging to Factor 2, regarding raising funds and recruiting people. The search for funding, establishing a partnership with researchers and companies, and attracting new members can help in the development of multicentric research studies with greater technological robustness. These resources guide the search for excellence in the research group’s knowledge area.

With regard to sufficient managerial competences, there was a higher occurrence in participants with master’s and doctorate degrees. MSs and PhDs have already gone through postgraduate studies in the stricto sensu modality in Nursing, which contributes to professor training by enabling the development of teaching skills, the ability to train new researchers and to manage research projects^17^.

The results showed that professors, leaders of research groups and post-PhDs are more likely to have sufficient managerial competences compared to undergraduate students, who are in the process of learning in research groups. The fact that professors, leaders of research groups and post-PhDs are encouraged to exercise skills for teaching and university management in their daily practice may have influenced the greater chance of presenting sufficient managerial competences compared to students who are still looking for skills development. As a result of the performance as a professor, researcher and manager, the higher education professor exercises different competences in each of these areas, since a single one does not cover the specificities required by each of these three fronts^2^.

Thus, professors are expected to mobilize different professional skills, including managerial competences to seek the efficiency and effectiveness of organizational management, through research and extension results and student learning. In this perspective, professors associate previous experiences, their academic trajectory, and even their life trajectory as justifications that permeate their competence repertoire^2^.

An international study on the essential competences of professors acting in the health area highlighted the competence for scientific research as the most expected by health education specialists in schools. The development of scientific research studies contributes for the teacher to develop flexibility, understanding the complexity of the training universities in the area of health and experimental pedagogical experience^18^.

A strategy described in the literature can assist Nursing professors in developing competences to leverage their careers, as well as leadership in Nursing research. Mentoring can be carried out individually or in groups and consists in the advising of experienced researchers to beginning researchers, through the transmission of information and support with a view to professional development. Mentoring has a positive influence on issues related to research productivity, such as: knowledge, funding, collaboration, and publications. This strategy, in addition to promoting the development of the research career, leadership and increased salaries, had repercussions on work relationships, on health, and on the well-being of Nursing professors^19^.

Although the researchers with higher education (professors, leaders and post-docs) working in the group are more likely to have sufficient managerial competences, when the stimulus for skills development is during the period of professional training, it points to better future results in terms of performance related to the managerial competences. The professionals who have acquired knowledge, in addition to their role, have greater capacity for innovation and knowledge applied to other areas^20^.

In the study results, the number of undergraduate students inserted in research groups stood out. The contact with research groups during undergraduation has a positive impact on the training of nurses who are critical and knowledgeable about research. The inclusion in research groups allows the undergraduate student to interact with postgraduate students and professors, to approach research projects, scientific initiation, and monitoring. This greater approximation with research also during the undergraduate course reflects in knowledge of the stricto sensu graduate program and, consequently, in the ease to enter these programs. Also during undergraduation, there is an approach to the construction of a research project, publication of articles, reading and discipline^21^.

In this sense, Nursing education with competence-based learning gives excellence to the teaching-learning process. This teaching modality has the advantage for teaching competences by the student: use of active methodologies; student accountability for their learning process; control of resources and curricular cohesion. These competences show the level of knowledge for the professional practice, which is essential for leading professional activity and for ensuring the quality of the professionals^22^.

A study carried out with undergraduate students identified the fundamental competences for training nurses in the three dimensions (knowledge, skill, and attitudes). Namely: health and nursing planning, information system, human, physical and material resources, change process, time management, leadership, decision-making, teamwork, supervision, conflict resolution, auditing, communication and continuing education^23^.

However, the representative number of undergraduate students in this study can reflect on the results in relation to the insufficiency/sufficiency of managerial competences, since there is an upward trend in these skills throughout the profession. A study carried out in the United States with 168 pharmacists, with the objective of identifying their managerial competences, showed that, while in graduation, 15% of the pharmacists reported a high degree of managerial competences and, after graduating, 57% presented a high degree of managerial competences, which have been developed throughout their careers^24^.

The data also indicated more chances of finding sufficient managerial competences in participants aged 46 to 55, compared to individuals up to 25 years old. Similarly, a study conducted in North American companies verified that the most successful companies are those created by founders over the age of 45. This result indicates that professional performance increases markedly with age, due to the experience acquired over the years^25^.

In each year of participation in the research group, the probability of having sufficient managerial competences increases by 18%. The research group promotes critical thinking, encourages its members to reflect on the praxis in Nursing and its diverse contexts, when relating practical experiences to university knowledge. This experience favors the development of personal and professional competences for the members of the research group^6^. In addition, the training process in the spaces of the research groups provides in-depth discussions on theoretical-methodological frameworks and epistemology, listed as one of the strategies that can even boost the dissemination of Nursing scientific knowledge at the international level^7^.

This way, research groups and postgraduation are important scenarios for the development of managerial competences. A study with North American professionals showed that most of them consider postgraduation as a facilitator to assist in the development of managerial competences^24^.

The intellectual dimension of a research group is made up by the association of original ideas and competences, especially by the association of knowledge of its members. Thus, the competences of the researchers must be identified as a way to manage and map the individual potentialities. In this way, the group can benefit from the knowledge produced to create and disseminate new products and services^1^.

The competence map is part of the architecture of a knowledge management system oriented to research groups, which can be used as a way to maximize results. This map consists of the prior establishment of essential competences identified at a certain level of knowledge in each of the members of research groups. Thus, after identifying these potentials, this knowledge is used to form research teams motivated to produce. This method, associated with interactive, group support, management and content tools, seeks satisfactory knowledge management in research groups^1^.

This study had limitations in presenting the individual competences of researchers from research groups. In this sense, it is necessary to expand discussions about organizational managerial competences, based on the definitions of competences of the groups and institutions, with monitoring and evaluation, using a competence management model and/or tools such as competence mapping.

Despite this, from the identification and mapping of the managerial competences of researchers, the study contributes to the advancement of scientific knowledge, pointing out new perspectives for people management with a focus on competence management. In this sense, the competences of the researchers are more easily identified and their performance in that field is enhanced to obtain better results in research.

## Conclusion

The managerial competences of participants in research groups in Nursing are mainly linked to the management of people and research results. As for sufficient managerial competences, there was a higher occurrence and a greater chance of showing sufficiency in the participants with higher academic degrees. Regarding the development of these competences, it was verified that the chance of presenting sufficient managerial competences increases with the time of participation in the research group, with the experience in research, and with age.

Thus, the study contributes to highlight the role played by the research groups for the development of managerial competences, since through it, activities that provide researchers with the exercise of these competences are linked. Although the process of learning competences is not always explicit within the actions of researchers and the research group, it is suggested to systematize this knowledge in order to enhance training. The definitions of organizational managerial competences of groups; tools for mapping competences, and a competence management model can assist in this process.
